# NMR resonance assignment of a ligand-binding domain of ephrin receptor A2

**DOI:** 10.1007/s12104-024-10211-4

**Published:** 2024-12-19

**Authors:** Konstantin S. Mineev, Santosh L. Gande, Verena Linhard, Sattar Khashkhashi Moghaddam, Harald Schwalbe

**Affiliations:** Institute for Organic Chemistry and Chemical Biology, Center for Biomolecular Magnetic Resonance (BMRZ), Johann Wolfgang Goethe University, Max-von-Laue-Str. 7, 60438 Frankfurt/Main, Germany

**Keywords:** Ephrin receptor, EphA2, Ephrin, NMR assignment, Ligand binding domain, Extracellular domain

## Abstract

Ephrin receptors regulate intercellular communication and are thus involved in tumor development. Ephrin receptor A2 (EphA2), in particular, is overexpressed in a variety of cancers and is a proven target for anti-cancer drugs. The N-terminal ligand-binding domain of ephrin receptors is responsible for the recognition of their ligands, ephrins, and is directly involved in receptor activation. Here, we report on the complete ^1^H, ^15^N and ^13^C NMR chemical shift assignment of EphA2 ligand binding domain that provides the basis for NMR-assisted drug design.

## Biological Context

Ephrin receptors (Eph) belong to the class of receptor tyrosine kinases, constituting their biggest subfamily, including 13 members in the case of humans. The receptors share an architecture of a type I membrane protein, with intracellular kinase and regulative SAM domains, a single-pass transmembrane domain and an extracellular part, consisting of two fibronectin type III domains, EGF-like domain, Sushi domain and a terminal ligand-binding domain (LBD) (Kullander and Klein 2002). Eph recognize their cognate ligands, ephrins, which are attached to the cell membrane either with GPI anchors (ephrins A) or by their own transmembrane domains (ephrins B) (Himanen 2012). Depending on the preferred type of the bound ligand, receptors are also classified into two groups: EphA and EphB. Eph are believed to be activated by self-clustering: binding of ephrin to the N-terminal ligand-binding domain of Eph triggers the rearrangement of the receptor cluster on the cell membrane, which, in turn, leads to the activation of their kinase activity (Singh et al. 2018; Shi et al. 2023). Eph receptors regulate the migration and mobility of various cells (Pasquale 2008; Kania and Klein 2016) and take part in angiogenesis and metastasis formation (Pasquale 2010), which makes them important pharmacological targets. EphA2, among others, is a proven target to treat the colorectal cancer (Tröster et al. 2023b). Therefore, structural investigations and Eph-targeted structure-guided drug design campaigns are highly important.

The three-dimensional structure of EphA2 is rather well explored. There are X-ray structures of the receptor kinase domains, both in the apo state and in complex with various ligands (Nowakowski et al. 2002; Heinzlmeir et al. 2016; Tröster et al. 2023a), an NMR structure of transmembrane domains in the dimeric state (Bocharov et al. 2010), several X-ray structures of truncated and non-truncated extracellular domains (Himanen et al. 2009, 2010; Seiradake et al. 2010; Himanen 2012), including those obtained in complex with ephrin ligands. NMR spectroscopy investigation of EphA2 kinase domains was also reported (Gande et al. 2016). On the other hand, it would be beneficial to conduct the NMR studies of extracellular LBD, to run NMR-assisted ligand screenings or structure-activity relationship investigations. The general possibility of obtaining the EphA2 LBD NMR spectra was previously reported (Wu et al. 2015), however, no chemical shift assignment or further structural study was conducted. Here, we present the complete NMR chemical shift assignment of EphA2 LBD, which provides the basis for further investigation.

## Methods and experiments

### Peptide synthesis

YSA peptide (H-YSAYPDSVPMMS-OH) was synthesized with free N- and C- termini using solid phase peptide synthesis on an ABI 433 A peptide synthesizer (Thermo Fisher). The resulting product was dissolved in water and further purified by reversed phase HPLC with C18 column material, followed by determination of purity and identity of the product by NMR and ESI-MS.

### Expression and purification of EphA2 LBD

The EphA2 LBD construct used in this study comprises the residues A24 to Q206 of human EphA2 (UniProt ID: P29317 ) and bears one single-point mutation, C201A, which was introduced to remove the unpaired cysteine residue that in the full-length protein forms a disulfide within the adjacent Sushi domain. The EPHA2 LBD gene construct, optimized for *E.coli*, was synthesized by GenScript Biotech (Netherlands) B.V. Subsequently, it was subcloned into the in-house modified pMALTEV vector, which harbors Maltose binding protein (MBP) to enhance the solubility and folding of the expressed fusion partner. pMALTEV vector is a modified version of the standard pMAL-c4x vector (NovoPro), that provides N-terminal hexahistidine residues to the MBP fusion protein and the tobacco etch virus (TEV) protease cleavage site after the 6XHis-MBP fusion tag. After the TEV cleavage, the resulting protein contained three additional amino acids at the N-terminus: (^21^GAM) due to the cloning. The expression was conducted in Shuffle^®^ T7 competent *E.coli* cells (New England BioLabs). Protein expressions were performed in 5 L Erlenmeyer flask either in either 15 N M9 (1 g/L ^15^NH_4_Cl) or 13 C, 15 N M9 (2 g/L ^13^C-D-Glucose, 1 g/L ^15^NH_4_Cl) medium with 100 µg/ml ampicillin. When the *A*_600_ of the cells reached 0.6–0.7, a cold shock was given to the cells by placing the flask in an ice bath for 20 min. Followed by induction with 75µM IPTG concentration and overnight expression at 18 °C with 120 RPM. Cells were harvested by centrifugation at 4,000 × g for 20 min and the cell pellets were stored at -70 °C until further processing.

The cell pellet was thawed and resuspended in 150 ml of lysis buffer or Ni-buffer A (50 mM Tris pH 8.2, 150 mM NaCl, 5 mM imidazole), supplemented with a protease inhibitor tablet (cOmplete™, Roche, Germany). Mechanical cell lysis was performed using a microfluidizer (Microfluidics M-100P) at a pressure of 15000 PSI for 4–6 cycles, while maintaining continuous cooling with ice. Eventually, the supernatant was loaded onto HisTrap HP columns (Cytiva, USA) that had been equilibrated with Ni-buffer A (following the manufacturer’s recommendations). Following the loading of the cell lysate, the HisTrap HP column was washed with 5% of Ni-buffer B (50 mM Tris pH 8.2, 150 mM NaCl, and 500 mM imidazole) with 10 column volumes and the elution of the target protein was performed with a linear gradient of Ni-buffer B from 5 to 100% in 10 column volumes. Fractions containing the target protein were pooled together and incubated with TEV-protease overnight at 4 °C, with simultaneous dialysis against Ni-buffer A. After the TEV cleavage, the dialyzed EPHA2 protein was applied once again on the HisTrap HP column to separate the EPHA2 LBD protein from the cleaved His-MBP fusion tag and TEV protease. The final purification of the EPHA2 LBD protein was achieved through HiLoad26/60 S75 size-exclusion chromatography using buffer C (50 mM Tris pH 8.2, 150 mM NaCl). The pure protein obtained from the Sect. 75 was concentrated using an Amicon concentrator with a regenerated cellulose membrane of 10 kDa cut-off, achieving concentrations of 2–10 mg/mL (0.1–0.5 mM) with the target protein yields of around 7 mg/L for M9 media. For NMR experiments, EPHA2 LBD was re-buffered to 25 mM NaPi pH 6.7).

**Fig. 1:**
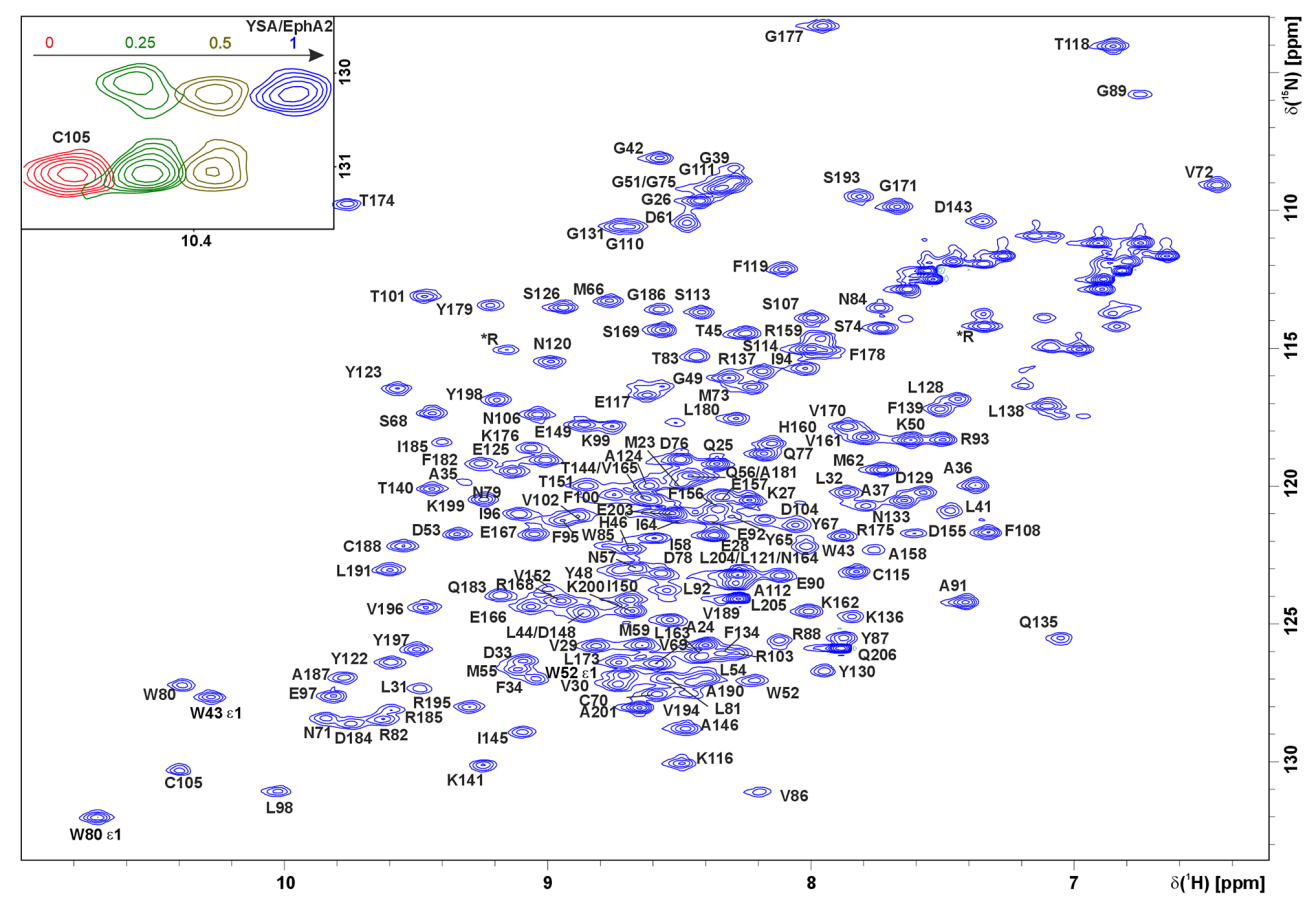
NMR assignment of EphA2 LBD.^1^H,^15^N-HSQC spectrum of ^13^C/^15^N-labeled EphA2 LBD, recorded at 303 K and 600 MHz in 25 mM NaPi pH 6.7, 5% D_2_O, 0.01% NaN_3_. Assignments of the backbone amide resonances are shown for each residue, an asterisk (*) denotes the signal of the protein side chains. Insert on top shows the overlay of ^1^H,^15^N-HSQC spectra (fragments, containing the C105 cross-peak), obtained after the addition of 0.25 (green), 0.5 (brown) and 1 (blue) equivalents of YSA peptide. The spectra are shifted horizontally with respect to each other for readability

The appropriateness of the EphA2 LBD folding was confirmed by titrating the ^15^N-labeled protein (300 µM) with 10 mg/mL stock solution of the YSA peptide, the well documented EphA2 LBD binder with low-micromolar affinity (Koolpe et al. 2002). Recorded NMR spectra revealed the clear high-affinity binding, which occurred in the slow exchange regime (Fig. 1), suggesting the proper folding and ligand-binding propensity of the protein.

### NMR experiments

Prior to NMR measurements, EphA2 LBD samples were supplied with 5% D_2_O, 0.1% NaN_3_ and 0.1 mM TSP for chemical shift referencing. The NMR samples (330 µL) were measured at either 298 or 303 K on Bruker Avance Neo 600 MHz spectrometer equipped with QCI cryoprobe. Triple resonance spectra were acquired using the BEST-TROSY pulse sequences (Favier and Brutscher 2011; Solyom et al. 2013), and non-uniform sampling of indirect dimensions was applied, if required. The backbone and side chain chemical shift assignments of EphA2 were performed using the standard triple resonance NMR experiments, with the assistance from 3D-HCCH-TOCSY and 3D NOESY-HSQC spectra. Aromatic sidechains were assigned using the 2D (Hb)Cb(CgCC)H (Löhr et al. 2007) and (H)CCH-COSY (Pervushin et al. 1998) experiments. Data were processed using TopSpin 4.4.0 software (Bruker BioSpin) and the spectra were analyzed using CARA 1.9.2 (Keller 2004) software. Spectra recorded with non-uniform sampling were processed in qMDD software using the iterative soft thresholding algorithm with virtual echo (Mayzel et al. 2014). The list of recorded NMR spectra is provided in Table 1.

**Table 1:** List of the NMR experiments with experimental conditions

Experiments	Labeling	Time domain data size [points]			Spectral width [ppm]			ns	Delay time [s]	Mixing time, ms	NUS %	T, K
		T1	T2	T3	F1	F2	F3					
BEST-TROSY	^13^C/^15^N	200	2024		35 (^15^N)	16.0 (^1^H)		8	0.3	-	-	303/298
^1^-^13^ C-HSQC		300	1024		80.0 (^13^C)	13.0 (^1^H)		2	1.0	-	-	303/298
HNCACB		256	116	2024	64.0 (^13^C)	31.0 (^15^N)	13.6 (^1^H)	24	0.3	-	40	298
HNCO		100	116	2024	14.0 (^13^C)	31.0 (^15^N)	13.6 (^1^H)	8	0.3	-	-	298
HN(CA)CO		100	116	2024	14.0 (^13^C)	31.0 (^15^N)	13.6 (^1^H)	32	0.3	-	40	298
HNCA		128	116	2024	26.0 (^13^C)	31.0 (^15^N)	13.6 (^1^H)	8	0.3	-	-	303/298
HN(CO)CA		128	116	2024	26.0 (^13^C)	31.0 (^15^N)	13.6 (^1^H)	8	0.3	-	-	298
H(C)CH-TOCSY		136	180	2048	10 (^1^H)	42.0 (^13^C)	13.6 (^1^H)	8	1.0	16.3	48	298
(H)CCH-TOCSY		220	128	1024	80.0 (^13^C)	42.0 (^13^C)	11.9 (^1^H)	8	1.0	10.9	45	303
NOESY-^15^N-HSQC		400	128	1024	11.4 (^1^H)	31.0 (^15^N)	11.4 (^1^H)	8	1.0	120	47	298
NOESY-^13^C-HSQC		220	128	1024	11.9 (^1^H)	42.0 (^13^C)	12.0 (^1^H)	16	1.0	120	40	303
NOESY-^13^Caro-HSQC		220	66	1024	11.9 (^1^H)	30.0 (^15^N)	12.0 (^1^H)	32	1.0	120	42	303
(H)CCH-COSY		142	48	2048	36.0 (^13^C)	36.0 (^13^C)	13.6 (^1^H)	8	1.0	-	-	303
(Hb)Cb(CgCC)H		94	1024		40.0 (^13^C)	10.9 (^1^H)		129	1.0	-	-	303

## Extent of assignment and data deposition

The EphA2 LBD protein consists of 186 amino acids, including six prolines, implying that 179 cross-peaks can be in theory observed in an ^1^H,^15^N-HSQC, while 172 amide cross-peaks were actually assigned, corresponding to 96% of maximally possible (Fig. 1B). Overall, we assigned 96.2% of the protein backbone, 97.6% of the aliphatic and 79.8% of aromatic side chain atoms. In addition, we identified the ^1^H and ^15^N resonances of eight asparagine and glutamine sidechains, three H𝜀_1_ cross-peaks of W43, W52 and W80, and one H𝜀 cross-peak of arginine sidechain (R195), based on the NOESY connectivities. Cross-peaks of following amide groups were not found: A22, E40, N60, T132, S153 and S154, due to the exchange-induced line broadening. Noteworthy, for A22, E40, N60 and T132 we assigned the signals of respective side chains.

**Fig. 2:**
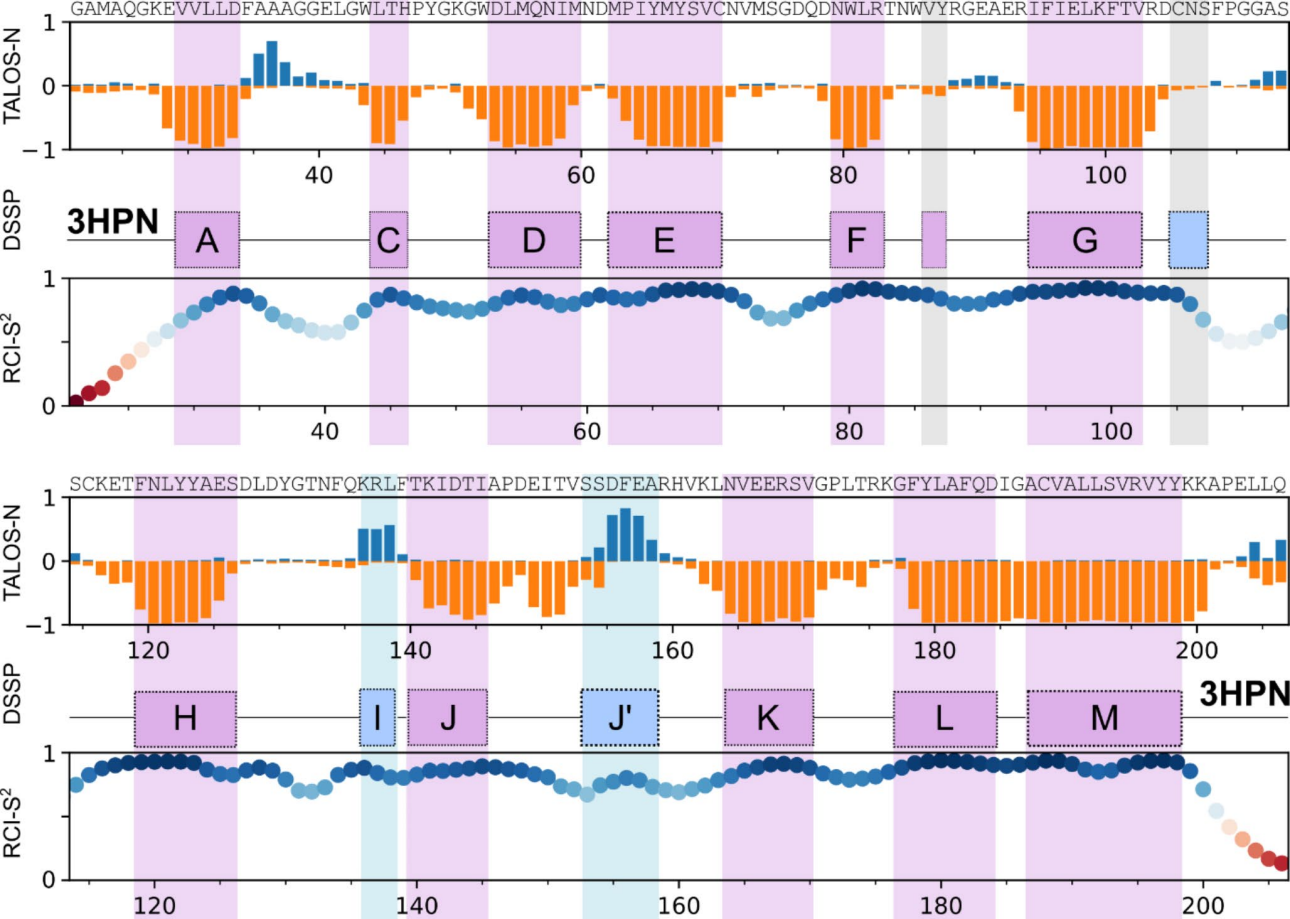
Results of the NMR chemical shift analysis. The chemical shift-derived propensity of the secondary structure (**TALOS-N**, is orange and negative for the β-structure and blue or positive for the ɑ-helix); the secondary structure of EphA2 LBD, according to the PDB ID 3HPN is shown for reference (**DSSP**, blue rectangles correspond to the helical structure and purple - to the extended conformation, grey rectangles denote the elements that are found in the structure, but are not present in solution, according to the NMR chemical shifts); and the order parameters of backbone amide groups, calculated based on the random coil chemical shift index (**RCI-S**^**2**^), are shown as a function of residue number. Elements of secondary structure are named according to the work (Himanen et al. 2009). Points are colored with respect to the RCI-S^2^ magnitude. The secondary structure shown is the consensus result of the DSSP (Kabsch and Sander 1983) and STRIDE (Heinig and Frishman 2004) packages

Figure 2 shows the secondary structure analysis of EphA2 LBD, performed using the TALOS-N software (Shen and Bax 2015). As a reference, we utilized the structure of EphA2 LBD with PDB ID 3HPN, which corresponds to a shorter ^28^E-^201^C construct (Himanen et al. 2009). According to the literature, EphA2 LBD has a jellyroll β-sandwich fold, comprising 11 β-strands and two short α-helices, which are traditionally named by letters from A to M. Additionally, the secondary structure analysis software, such as STRIDE (Heinig and Frishman 2004) or DSSP (Kabsch and Sander 1983), detects a short β-strand in the F-G loop and a 3–10 helix in the G-H loop. Altogether this fold is perfectly reproduced in the NMR chemical shifts data. All the major elements of secondary structure are clearly observed, except for the short strand and 3–10 helix in the loops, mentioned above. Besides, one could state that the β-strand J in solution becomes elongated, compared to the X-ray structure, which could be the consequence of either construct length or crystallization. According to the random coil chemical shift index (Berjanskii and Wishart 2005), the first eight and last six residues of EphA2 LBD construct are disordered.

In conclusion, here we report an NMR chemical shift assignment of the ligand binding domain of Ephrin receptor A2. The assignment is almost complete and is in good agreement with the spatial structure of EphA2, reported previously. The quality of the data and completeness of the assignment allow exploiting our data in future ligand screening campaigns and structural studies.

## Data Availability

NMR resonance assignments have been deposited to the BMRB under the accession code: 52671.

## References

[CR1] Berjanskii MV, Wishart DS (2005) A Simple Method To Predict Protein Flexibility Using Secondary Chemical Shifts. J Am Chem Soc 127:14970–14971. 10.1021/ja054842f 10.1021/ja054842f 16248604

[CR2] Bocharov EV, Mayzel ML, Volynsky PE, et al (2010) Left-handed dimer of EphA2 transmembrane domain: Helix packing diversity among receptor tyrosine kinases. Biophys J 98:881–889. 10.1016/j.bpj.2009.11.008 10.1016/j.bpj.2009.11.008 PMC2830432 20197042

[CR3] Favier A, Brutscher B (2011) Recovering lost magnetization: polarization enhancement in biomolecular NMR. J Biomol NMR 49:9–15. 10.1007/s10858-010-9461-5 10.1007/s10858-010-9461-5 21190063

[CR4] Gande SL, Saxena K, Sreeramulu S, et al (2016) Expression and Purification of EPHA2 Tyrosine Kinase Domain for Crystallographic and NMR Studies. Chembiochem Eur J Chem Biol 17:2257–2263. 10.1002/cbic.201600483 10.1002/cbic.201600483 27685543

[CR5] Heinig M, Frishman D (2004) STRIDE: a web server for secondary structure assignment from known atomic coordinates of proteins. Nucleic Acids Res 32:W500–W502. 10.1093/nar/gkh429 10.1093/nar/gkh429 PMC441567 15215436

[CR6] Heinzlmeir S, Kudlinzki D, Sreeramulu S, et al (2016) Chemical Proteomics and Structural Biology Define EPHA2 Inhibition by Clinical Kinase Drugs. ACS Chem Biol 11:3400–3411. 10.1021/acschembio.6b00709 10.1021/acschembio.6b00709 27768280

[CR7] Himanen JP (2012) Ectodomain structures of Eph receptors. Semin Cell Dev Biol 23:35–42. 10.1016/j.semcdb.2011.10.025 10.1016/j.semcdb.2011.10.025 22044883

[CR8] Himanen JP, Goldgur Y, Miao H, et al (2009) Ligand recognition by A-class Eph receptors: crystal structures of the EphA2 ligand-binding domain and the EphA2/ephrin-A1 complex. EMBO Rep 10:722–728. 10.1038/embor.2009.91 10.1038/embor.2009.91 PMC2727437 19525919

[CR9] Himanen JP, Yermekbayeva L, Janes PW, et al (2010) Architecture of Eph receptor clusters. Proc Natl Acad Sci U S A 107:10860–10865. 10.1073/pnas.1004148107 10.1073/pnas.1004148107 PMC2890748 20505120

[CR10] Kabsch W, Sander C (1983) Dictionary of protein secondary structure: pattern recognition of hydrogen-bonded and geometrical features. Biopolymers 22:2577–2637. 10.1002/bip.360221211 10.1002/bip.360221211 6667333

[CR11] Kania A, Klein R (2016) Mechanisms of ephrin-Eph signalling in development, physiology and disease. Nat Rev Mol Cell Biol 17:240–256. 10.1038/nrm.2015.16 10.1038/nrm.2015.16 26790531

[CR12] Keller RLJ (2004) The Computer Aided Resonance Assignment Tutorial. CANTINA Verlag, Goldau, Switzerland

[CR13] Koolpe M, Dail M, Pasquale EB (2002) An Ephrin Mimetic Peptide That Selectively Targets the EphA2 Receptor *. J Biol Chem 277:46974–46979. 10.1074/jbc.M208495200 10.1074/jbc.M208495200 12351647

[CR14] Kullander K, Klein R (2002) Mechanisms and functions of eph and ephrin signalling. Nat Rev Mol Cell Biol 3:475–486. 10.1038/nrm856 10.1038/nrm856 12094214

[CR15] Löhr F, Hänsel R, Rogov VV, Dötsch V (2007) Improved pulse sequences for sequence specific assignment of aromatic proton resonances in proteins. J Biomol NMR 37:205–224. 10.1007/s10858-006-9128-4 10.1007/s10858-006-9128-4 17237975

[CR16] Mayzel M, Kazimierczuk K, Orekhov VY (2014) The causality principle in the reconstruction of sparse NMR spectra. Chem Commun Camb Engl 50:8947–8950. 10.1039/c4cc03047h 10.1039/c4cc03047h 24975496

[CR17] Nowakowski J, Cronin CN, McRee DE, et al (2002) Structures of the cancer-related Aurora-A, FAK, and EphA2 protein kinases from nanovolume crystallography. Struct Lond Engl 1993 10:1659–1667. 10.1016/s0969-2126(02)00907-3 10.1016/s0969-2126(02)00907-3 12467573

[CR18] Pasquale EB (2008) Eph-ephrin bidirectional signaling in physiology and disease. Cell 133:38–52. 10.1016/j.cell.2008.03.011 10.1016/j.cell.2008.03.011 18394988

[CR19] Pasquale EB (2010) Eph receptors and ephrins in cancer: bidirectional signalling and beyond. Nat Rev Cancer 10:165–180. 10.1038/nrc2806 10.1038/nrc2806 PMC2921274 20179713

[CR20] Pervushin K, Riek R, Wider G, Wüthrich K (1998) Transverse Relaxation-Optimized Spectroscopy (TROSY) for NMR Studies of Aromatic Spin Systems in 13 C-Labeled Proteins. J Am Chem Soc 120:6394–6400. 10.1021/ja980742g

[CR21] Seiradake E, Harlos K, Sutton G, et al (2010) An extracellular steric seeding mechanism for Eph-ephrin signaling platform assembly. Nat Struct Mol Biol 17:398–402. 10.1038/nsmb.1782 10.1038/nsmb.1782 PMC3672960 20228801

[CR22] Shen Y, Bax A (2015) Protein structural information derived from NMR chemical shift with the neural network program TALOS-N. Methods Mol Biol Clifton NJ 1260:17–32. 10.1007/978-1-4939-2239-0_2 10.1007/978-1-4939-2239-0_2 PMC4319698 25502373

[CR23] Shi X, Lingerak R, Herting CJ, et al (2023) Time-resolved live-cell spectroscopy reveals EphA2 multimeric assembly. Science 382:1042–1050. 10.1126/science.adg5314 10.1126/science.adg5314 PMC11114627 37972196

[CR24] Singh DR, Kanvinde P, King C, et al (2018) The EphA2 receptor is activated through induction of distinct, ligand-dependent oligomeric structures. Commun Biol 1:15. 10.1038/s42003-018-0017-7 10.1038/s42003-018-0017-7 PMC6123813 30271902

[CR25] Solyom Z, Schwarten M, Geist L, et al (2013) BEST-TROSY experiments for time-efficient sequential resonance assignment of large disordered proteins. J Biomol NMR 55:311–321. 10.1007/s10858-013-9715-0 10.1007/s10858-013-9715-0 23435576

[CR26] Tröster A, DiPrima M, Jores N, et al (2023a) Optimization of the Lead Compound NVP-BHG712 as a Colorectal Cancer Inhibitor. Chem Weinh Bergstr Ger 29:e202203967. 10.1002/chem.202203967 10.1002/chem.202203967 PMC10133194 36799129

[CR27] Tröster A, Jores N, Mineev KS, et al (2023b) Targeting EPHA2 with Kinase Inhibitors in Colorectal Cancer. ChemMedChem 18:e202300420. 10.1002/cmdc.202300420 10.1002/cmdc.202300420 PMC10843416 37736700

[CR28] Wu B, Wang S, De SK, et al (2015) Design and Characterization of Novel EphA2 Agonists for Targeted Delivery of Chemotherapy to Cancer Cells. Chem Biol 22:876–887. 10.1016/j.chembiol.2015.06.011 10.1016/j.chembiol.2015.06.011 PMC4515144 26165155

